# Step-wise evolution of complex chemical defenses in millipedes: a phylogenomic approach

**DOI:** 10.1038/s41598-018-19996-6

**Published:** 2018-02-16

**Authors:** Juanita Rodriguez, Tappey H. Jones, Petra Sierwald, Paul E. Marek, William A. Shear, Michael S. Brewer, Kevin M. Kocot, Jason E. Bond

**Affiliations:** 1 Department of Biological Sciences, Auburn University, Auburn, AL 36849 USA; 2 CSIRO, Australian National Insect Collection, Canberra, ACT 2601 Australia; 3 Department of Chemistry, Virginia Military Institute, Lexington, VA 24450 USA; 4 Zoology Department, The Field Museum, Chicago, IL 60605 USA; 5 Department of Entomology, Virginia Tech, Blacksburg, VA 24061 USA; 6 Biology Department, Hampden-Sydney College, Farmville, VA 23943 USA; 7 Department of Biology, East Carolina University, Greenville, NC 27858 USA; 8 Department of Biological Sciences, University of Alabama, Tuscaloosa, AL 35487 USA

## Abstract

With fossil representatives from the Silurian capable of respiring atmospheric oxygen, millipedes are among the oldest terrestrial animals, and likely the first to acquire diverse and complex chemical defenses against predators. Exploring the origin of complex adaptive traits is critical for understanding the evolution of Earth’s biological complexity, and chemical defense evolution serves as an ideal study system. The classic explanation for the evolution of complexity is by gradual increase from simple to complex, passing through intermediate “stepping stone” states. Here we present the first phylogenetic-based study of the evolution of complex chemical defenses in millipedes by generating the largest genomic-based phylogenetic dataset ever assembled for the group. Our phylogenomic results demonstrate that chemical complexity shows a clear pattern of escalation through time. New pathways are added in a stepwise pattern, leading to greater chemical complexity, independently in a number of derived lineages. This complexity gradually increased through time, leading to the advent of three distantly related chemically complex evolutionary lineages, each uniquely characteristic of each of the respective millipede groups.

## Introduction

One of the most significant events in the evolution of early life on planet Earth took place in the middle Silurian —roughly 423 million years ago— with the origin of the first land animals^1,2^, triggering the greatest documented expansion of species-level diversity^3^. Because arthropods are the oldest land animals, and the most species-rich group ever to inhabit the planet, the evolution of chemical defenses largely employed now against predators, likely played a major role in their early diversification. Traits shaped by co-evolutionary interactions between species, such as defensive mechanisms, are often complex morphological/physiological systems; how these systems originated through the action of mutation, drift and natural selection is a central question in evolutionary biology^4^. Despite the complexity and importance of arthropod chemical defense systems, most studies have focused on understanding the signaling of defense against predators^5,6^ rather than the evolution of chemical defenses themselves^7^, therefore their origin and mode of evolution is yet fully understood. Here we investigate the evolution of complex chemical defense systems in the arthropod class Diplopoda (millipedes) using an interdisclipinary approach that employs analytical chemistry and modern genomics-based approaches to generate an evolutionary, phylogenetic framework to investigate chemical defense evolution across earth’s oldest known terrestrial animal group.

Simply stated, the central paradigm for the evolution of complexity is that natural selection drives a gradual elaboration towards multi-part complex traits, with intermediate steps between simple and complex structures^8–10^. Such a stepwise progression towards complexity is not widely accepted because of shortcomings associated with the assumption that evolution proceeds from simple to complex in light of the many documented cases where evolution results in loss or reduced functionality of structures^10^. Nonetheless, step-wise evolution from simple to complex systems has been observed at the anatomical, behavioral, molecular and metabolic level^11–14^. At the metabolic level, for example, the addition of reaction pathways has been observed to provide a stepping-stone model-like approach toward complex metabolic features in the face of nascent evolutionary pressures^14^.

**Figure 1:**
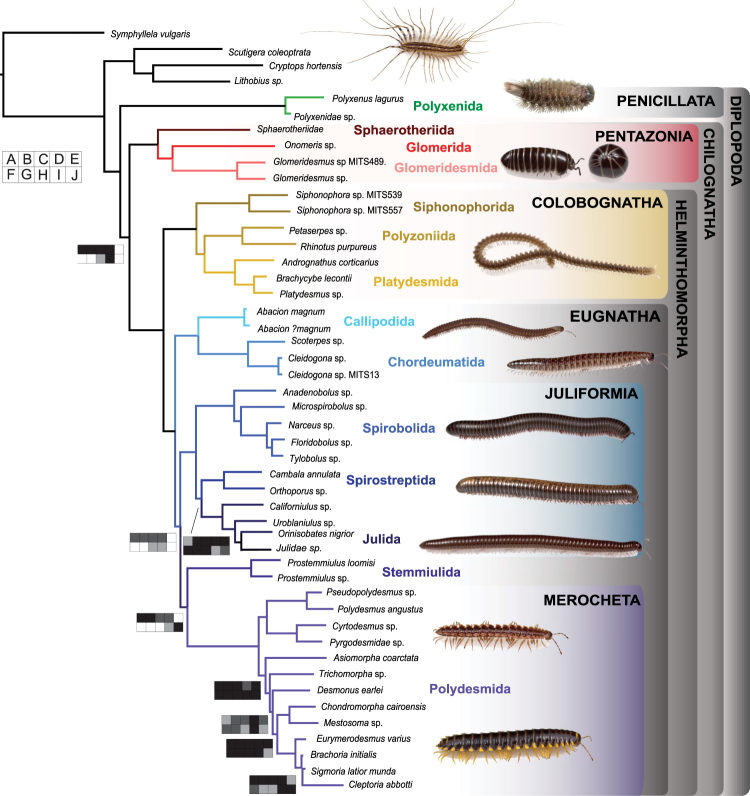
Maximum likelihood tree with branch lengths obtained from analysis of matrix M1. Tree is rooted with *Symphylella vulgaris*. Bootstrap support (BS) values for all analyses are summarized. BS values not indicated are 100%. Boxplots indicate bootstrap value ranges for each node across all analyses (**A**–**J**). Black boxes represent 90–99% BS, dark grey boxes 70–89% BS, light grey boxes BS less than 69% BS, and white boxes where the node whas not recovered for the analysis. (**A**) ExaML optimal ML tree for matrix M1. (**B**) ExaML optimal ML tree for matrix M2. (**C**) ExaML optimal ML tree for matrix M3. (**D**) ExaML optimal ML tree for matrix M4. (**E**) ASTRAL greedy consensus tree for matrix M1. (**F**) ASTRAL greedy consensus tree for matrix M2. (**G**) ASTRAL greedy consensus tree for matrix M3. (**H**) ASTRAL greedy consensus tree for matrix M4. (**I**) ExaML optimal ML tree for matrix M5. (**J**) ExaML optimal ML tree for matrix M6.

**Figure 2:**
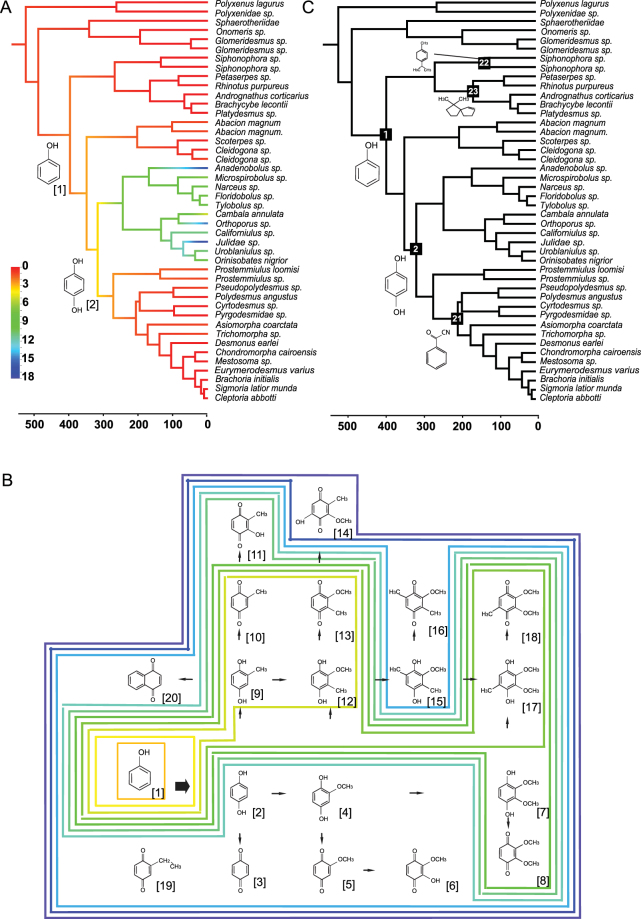
A chronogram for millipedes showing ancestral reconstruction for chemical complexity. (**A**) Benzoquinone chemical evolution and chemical pathway. Molecular structures on nodes of represent phenol origin and ancestral hydroquinone. Chemical evolution mapped as a continuous character. Colors on phylogeny branches represent number of chemicals produced (**A**) and correspond to colors encircling molecular structures in the chemical pathway (**B**). (**B**) Hypothetical chemical pathway representing the diversity of benzoquinone defense from their corresponding hydroquinone (modified from^79^). (**C**) Ancestral chemical ML reconstruction summarized from Figures S1.3–S1.5. Chemical with highest probability (>75%) is shown on the first node reconstructed. [1] Phenol, [2] Hydroquinone, [3] 1,4-Benzoquinone, [4] 2-Methoxyhydroquinone, [5] 2-Methoxy-1,4-benzoquinone, [6] 2-Methoxy-3-hydroxy-1,4-benzoquinone, [7] 2,3-Dimethoxyhydroquinone, [8] 2,3-Dimethoxy-1,4-benzoquinone, [9] 2-Methylhydroquinone, [10] 2-Methyl-1,4-benzoquinone, [11] 2-Methyl-3-hydroxy-1,4-benzoquinone, [12] 2-Methoxy-3-methylhydroquinone, [13] 2-Methoxy-3-methyl-1,4-benzoquinone, [14] 2-Methoxy-3-methyl-5-hydroxy-1,4-benzoquinone, [15] 2-Methoxy-3,6-dimethylhydroquinone, [16] 2-Methoxy-3,6-dimethyl-1,4-benzoquinone, [17] 2,3-dimethoxy-5-methylhydroquinone, [18] 2,3-dimethoxy-5-methyl-1,4-benzoquinone, [19] 2-Ethyl-1,4-benzoquinone, [20] Naphthoquinone, [21] Benzoil cyanide, [22] alpha-terpinene, [23] Polyzonimine.

Here we study the chemicals and pathways involved in millipede defense systems (Diplopoda), their phylogenetic comparative distributions, and hypothesized mode of evolution. Millipedes (class Diplopoda, Fig. 1) are the oldest known fully terrestrial animal group^1,15^ and are the most diverse class within the Myriapoda with approximately 12,000 known species worldwide and between 3,000 to 80,000 remaining to be described^16^. Furthermore, millipedes are ubiquitous in forest ecosystems, performing an important role as soil detritivores^17,18^. All but five millipede orders have repugnatorial glands that secrete chemical defenses when disturbed by predators^19^. These chemicals belong to at least eight molecule types (i.e., 1,4-benzoquinones, phenols, hydrogen cyanide, quinazolinones, and alkaloids, see Fig. 2)^20^. The most complex chemical system within millipedes—in terms of diversity of chemical structure and associated anatomy—is found in Juliformia (see Fig. 1) with the production of benzoquinones (Fig. 2, green blue clade). Juliforms produce a variety of other compounds not involved in the benzoquinone pathway like fatty acid esters and aliphatic compounds^20^. This system ranges from families that produce two chemical molecules (e.g. Nemasomatidae, Parajulidae, Cambalidae, see SI Appendix, Table S1.5) to families that can produce over 16^20,21^. As new molecule types are discovered and attribute greater chemical complexity to individual millipede families, there is clearly an observed pattern of a positive directional increase within chemical pathways through evolutionary time (Fig. 2). For example, phenol has been found to be the most widely shared chemical type, being produced by some species in six of the seven eugnathan orders in Callipodida, Spirobolida, Spirostreptida, Julida, Stemmiulida and Polydesmida (Figs 1 and 2). Studies on the evolution of phenol secretions in other arthropod groups have recently produced surprising results suggesting a single origin for its production, ancestral to quinones^22,23^, which indicates that phenols are liklely to be quinone precursors, and the first step in the benzoquinone pathway (Fig. 2). Studies focusing on patterns of chemical production in millipedes have led to the same hypothesis^20,24^, but in the absence of a well-sampled phylogenetic framework, proper ancestral character mapping methods and models of character evolution.

To determine if millipede generated phenols are ancestral to benzoquinones and to accurately model the sequence of events leading to the evolution of chemical systems in millipedes, a robust phylogeny and a reliable timescale of diversification are needed. However, the age and relationships between the main millipede lineages remain at this time controversial. The fossil record of Paleozoic millipedes is sparse^1,25^ and suggests that the origin of crown-group millipedes (i.e., most recent common ancestor of all living millipedes) is dated at least at 423 Ma^1^. Molecular divergence dating studies estimate an older origin for millipedes^15,26–28^. Moreover, various attempts have been made at elucidating the phylogenetic relationships among the orders^15,19,29,30^. The ordinal relationships and monophyly of higher taxa are still debated because reconstructions using morphological and molecular data have low support or are based on incomplete taxon sampling. Molecular phylogenetic analyses have generated some surprising results, including the paraphyly of Nematophora, and the placement of Stemmiulida as sister to Polydesmida^15,27^. These past results have key implications for understanding chemical evolution, suggesting that, because of their near-universality in Eugnatha, phenolic compounds are the oldest defense chemicals produced by eugnathan millipedes. Consequently, the conflict between morphological and traditional (Sanger-based) molecular data provides little in the way of reliable answers to some of these long-established evolutionary questions.

To address these issues, we obtained transcriptomic data for 44 millipede taxa from 14 orders to reconstruct the most complete phylogenomic hypothesis for millipedes ever to date. From the literature and newly analyzed data (first reported here), we then determined the chemical composition for six millipede species (Table S1.2) and added information of published chemical records from Shear^20^ for another 155 species from all but one of the chemically defended orders. For Juliformia we put together data for a total of 35 species, including six newly analyzed. These data indicate the highest diversity of chemical defense molecules occurs in the benzoquinone pathway, produced by the superorder Juliformia. One family from each of the three orders in this group produces more than 15 varieties of quinones (Fig. 2).

Based on a newly derived phylogenetic framework and a synthesis of all the chemical data available to us at this time, the main goal of our study is to test the hypothesis of step-wise evolution from simple to complex^8–10^ in arthropod chemical defense systems using a phylogenetic comparative approach by asking (i) did benzoquinone and cyanogenic production evolve after the ability to produce phenols in eugnathan millipedes and (ii) were the benzoquinone defense systems produced in a step-wise fashion from simple (few molecule types) to complex (many molecule types)?

## Results and Discussion

### Phylogenetic relationships among main millipede lineages

Although some progress has been made towards inferring phylogenetic relationships among millipede orders^15,19,27–30^, ordinal relationships and monophyly of major clades remain largely unresolved. The most widely supported hypothesis is that Penicillata (bristly millipedes) diverged from Chilognatha early in the history of the group. This hypothesis was traditionally based on the presence of “primitive” characters in Penicillata^29,31–35^ and has phylogenetic support from both morphological and molecular data^27,29^. Within Chilognatha, the monophyly of Pentazonia (Glomerida, Sphaerotheriida, Glomeridesmida) has been largely supported, but the monophyly of the Oniscomorpha (Glomerida, Sphaerotheriida), a clade based on the ability to form a sphere and the presence of an enlarged second tergite and anal shield^29,36^, remains controversial because morphological data support the grouping^29,31^, whereas molecular and combined datasets render it paraphyletic^19,27^. These results suggest the ability to roll into a sphere has been secondarily lost in the Glomeridesmida. Volvation is documented in four (Sphaerotheriida, Glomerida, Polyzoniida, Polydesmida) of the 16 millipede orders^37^ and has evolved repeatedly in many soil-dwelling arthropods. The large clade Helminthomorpha has been proposed based on the presence of gonopods on the seventh body ring in males^29^ and by the serial lateral repugnatory glands^38,39^ the monophyly of which has been supported by all phylogenetic analyses to date (15, 19, 27–29). The monophyly of the Colobognatha and its sister-group relationship to the Eugnatha has variously been disputed (as summarized by Hoffman^36^, but both have been supported by all phylogenetic analyses^15,27,29^). A placement of the Colobognatha as a tentative sister to the Polydesmida was considered spurious by the authors (19), suggesting further data collection being required. Another result that affects the inference of phenol evolution is the putative monophyly of Eugnatha –supported in all analyses except a single combined morphological/molecular dataset^19^. All Eugnatha, except for the order Chordeumatida, have families that produce phenols^40^. This suggests a single origin before benzoquinone and cyanogenic production. One of the most controversial groupings in previous classifications, the Nematophora^29,31^, was established on the basis of the presence of pre-anal spinnerets^41^, uniting the orders Callipodida, Chordeumatida and Stemmiulida. The non-monophyly of the Nematophora had been recovered in a total evidence analysis^19^ and recent phylogenomics studies^15,27^. The monophyly of the recently proposed “eighth gonopod clade” (ref.^42^, postulated to contain the orders Callipodida, Chordeumatida, Polydesmida and Stemmiulida) is not confirmed by our current analysis (Fig. 1). The spinnerets of Stemmiulida are morphologically distinct from those of Callipodida and Chordeumatida, but those of Polydesmida are virtually identical to Callipodidan spinnerets, suggesting that the form of the spinnerets in Stemmiulida is an autapomorphy, and that spinnerets have been lost in Juliformia^43^. It is uncertain whether the modified setae and the epiproct of the Stemmiulida are actually functioning as silk-producing organs^43^.

To produce a well-sampled phylogeny for the Diplopoda we generated transcriptomes and identified orthologs for 44 taxa across 26 families and 14 orders (average *de novo* assembled transcripts per species 49,523; average length 773), and combined these with orthologs from 15 published transcriptomes from ingroup and outgroup taxa (SI Appendix, Table S1.1). Our concatenated analyses of matrices M1-M4 largely recover lineages supported by earlier phylogenetic studies^29,30^. Penicillata is placed as sister to all remaining millipedes, branching off the earliest among the orders. Morphological characters supporting this early branching are the lack of gonopods^32^, uncalcified cuticle, indirect sperm transfer, well-developed body musculature^33^, serrated bristles for defense, and hemianamorphosis^34^ in Penicillata. As in most previous analyses the monophyly of the Chilognatha is recovered, and supported by several morphological characters^38,39^. Whereas Pentazonia is monophyletic, the monophyly of the Oniscomorpha (Glomerida, Sphaerotheriida) is rejected in all of our analyses, supporting the loss of the ability to roll into a sphere in Glomeridesmida. Helminthomorpha is monophyletic, supported morphologically by the presence of serial lateral pairs of defense glands (lost in Chordeumatida and Siphoniulida) tracheal characters^38,39^, and the adult being composed of at least 21 body rings^29^ although many polydesmids comprise 20 segments. Within Helminthomorpha, a monophyletic Colobognatha is sister to a monophyletic Eugnatha. Colobognatha is characterized by simple leg-like gonopods —as well as a number of autapomorphies proposed by Enghoff^29^: eight legs anterior to the male gonopods, gnathochilarial palps absent, first larval stadium with four pairs of legs, eggs protected by adults by curling around them, defense glands elongate-subtubular. It should be noted that morphological variability within millipedes has not been thoroughly examined across a wide spectrum of taxa^19^. Within Colobognatha the placement of Siphonophorida had only been established by a combined dataset^19^, where it was sister to Siphonocryptida + Platydesmida. We present here the first transcriptome data for this order and support its sister relationship to Polyzoniida + Platydesmida (Siphonocryptida is not included in our analysis). Eugnatha is supported by the results in each of our analyses, and is characterized morphologically by the pleurites fused to tergites, subspherical defense glands^29^, seven pairs of legs in front of the gonopods and an ontogenetic development strikingly different from the ontogeny of the colobognathan gonopods, suggesting non-homology between the colobognathan and eugnathan gonopods^30^. Within Eugnatha, a clade formed by Callipodida sister to Chordeumatida is sister to all remaining eugnaths. This sister relationship had been proposed by all earlier phylogenetic reconstructions (19, 28, 29) and long established morphologically by the special structure of the Tömösváry organ^34^. The monophyly of remaining eugnaths, however, has low support in ASTRAL analyses with high amounts of missing data, and in the most phylogenetic informative dataset (M6). This is not surprising given the sensitivity of ASTRAL to non-random missing data^44^ and the selection of high informativeness to certain time-frames, which could affect informativeness at other points in the phylogeny (see Materials and Methods). Juliformia is herein widely supported as monophyletic and Nematophora is polyphyletic. Juliformia has been established based on the sternites fused to pleurotergites, second larval stadium with seven pairs of legs, collum enlarged, defensive secretions with benzoquinones, and spermatozoa with pseudoperforatorium^29^. Although the monophyly of Juliformia has never been controversial, the relationships between its three orders had shown divergent results through the years, suggesting all possible combinations of relationships^19,31^. Our results support the sister relationship between Spirostreptida and Julida, albeit with lower support values for the supermatrix and informativeness analyses. Within Spirostreptida, the position of Cambaloidea had been controversial^30^. All our analyses include this suborder within Spirostreptida. Enghoff *et al*.^34^ suggested the validity of the sister relationship Polydesmida + Juliformia, and referred to it as the “ring forming millipedes”. Our topology infers Juliformia as sister to a clade formed by Stemmiulida and Polydesmida, which is supported by concatenated and ASTRAL analyses with low missing data. Again, this could be explained by the impact of missing data on the accuracy of ASTRAL reconstructions^44^. The lack of complete body rings in Stemmiulida in light of these relationships suggests a possible reversal of the formation of complete body rings in this order, as well as another reversal in a family of the order Julida, the Nemasomatidae^45^. Another –less parsimonious– option could be two separate, independent origins for ring formation in Polydesmida and Julimorpha (Fig. 1). Orders not included in this analysis are Siphoniulida and Siphonocryptida. The latter has only been included in a combined dataset and proposed as sister to Platydesmida^19^. The relationships of Siphoniulida are still obscure. Enghoff^29^ proposed that it should be considered a subordinate taxon within a juliformian or colobognath order. Despite the discovery of new specimens of this enigmatic group, phylogenetic analyses produced equivocal results, and therefore it is still placed as Helminthomorpha *incertae sedis*^30^. Divergence time estimation results suggest an origin for Diplopoda ca. 467 Ma (95% HPD: 424–531). This age is much earlier than suggested by the fossil record, but the youngest bound of the 95% HPD coincides with the oldest diplopod fossil recorded^1^.

### Chemical evolution

To estimate divergence times onto the millipede phylogeny we analyzed a reduced dataset of 38 partitions using a relaxed molecular clock and eight calibration points based on fossil data (SI Appendix, Table S1.4). We also produced a chronogram for the complete phylogeny using dates produced by the aforementioned analysis in a Penalized Likelihood framework^46,47^. Chemical composition was determined from published records and chemical analyses (SI Appendix, Table S1.2). Chemicals grouped into quinazolinone alkaloids, heterocyclic nitrogen-containing compounds, terpenes, benzoquinones-hydroquinones and phenol, and cyanogenics were mapped as separate characters (Appendix S1, Figures S1.2–S1.5). Phenol and benzoquinone production were mapped as a single discrete character to determine if phenol production was the ancestral condition (Appendix S1, Figure S1.2). Benzoquinone complexity was mapped onto the phylogeny as a continuous character to identify the directionality of chemical evolution (Fig. 2A,B). Our results indicate that the origin of phenol production occurred once during the evolution of millipedes at 315 Ma when Callipodida and Chordeumatida diverged from remaining eugnaths or twice with independent origin in Callipodida and the MRCA of Juliformia, Stemmiulida and Polydesmida, 275 Ma (Appendix S1, Figure S1.2). Our analyses support the origin of phenol production preceding benzoquinone production (Fig. 2A,C), and this chemical may be the first produced by millipede ancestors in the Silurian, and could be the oldest evidence of chemical production by land animals. Moreover, phenol production is the ubiquitous condition within Juliformia. The same pattern of phenol-benzoquinone evolution had been reported for Opiliones^22^ indicating that quinones could be produced by phenol para-oxidation and constitute —in many cases— an extension of the phenol biochemical pathway^22,23^. For millipedes it had been shown that phenol taxonomic distribution was patchy in Julida with multiple independent losses previously hypothesized^24^. Our reconstruction shows that phenol as a final product was lost multiple times in Juliformia (Fig. 2), and supports the hypothesis that phenol production is not essential for quinone production, further showing that other precursors may be involved in the pathway and that chemical complexity in millipedes was retained even in the absence of phenol production. Feeding experiments in millipedes have demonstrated that aromatic amino acids such as tyrosine may be precursors for phenol production mediated by tyrosine-phenol-lyase, an enzyme so far only recorded for Enterobacteriaceae^48^. Nonetheless, benzoquinone production in the absence of aromatic amino acids has been reported through the acetate pathway in millipedes and beetles with methionine as a precursor^49,50^. Even in the absence of phenols, the benzoquinone end product seems to require a hydroquinone precursor. This is supported by the presence of benzoquinones and their corresponding hydroquinones in the majority of our chemical samples. Our character reconstruction results show the ancestral condition for all Juliformia was the production of the precursor 2-methoxy-3-methylhydroquinone (Fig. 2A). Therefore, benzoquinone production originated by the ability to oxidize hydroquinones into their corresponding benzoquinone as had been hypothesized by earlier studies on arachnids^23^. The production of ethyl-benzoquinones is here only reported for Julidae, Rhinocricidae and Spirostreptidae, which are the three most chemically complex families. Recent analyses for cave-dwelling millipedes, demonstrate that ethyl-benzoquinone production had multiple independent origins, but it is not correlated to cave-dwelling^21^. Our results demonstrate independent origins even at the family level.

The retention of phenol production in Polydesmida lineages is not directly involved in the biosynthesis of their main aromatic defense chemicals (e.g., mandelonitrile, benzaldehyde, benzoyl cyanide), as these have been shown to derive from aromatic amino acids like phenylalanine^51^, nonetheless phenol has been proposed to be involved in maintaining the acidity needed for their biosynthesis^52^, and is the main chemical defense of immatures even when adults produce cyanogenics. This may suggest the secondary loss of cyanogenic production in some Polydesmida, probably due to the high cost of producing cyanogenics from amino acids^53^. Phenols are not directly involved in the production of other molecule groups such as quinazolinone alkaloids, or heterocyclic nitrogen-containing compounds^20^, which may indicate that these compounds arose as defenses independently.

Our results indicate that complexity arose from a simple ancestral condition of phenol production at least when Juliformia diverged from Polydesmida + Stemmiulida, and produced complex phenotypes convergently in three separate lineages, each from a different juliform order. Nonetheless, the ancestral complexity was also lost separately in two lineages. A central challenge in evolutionary biology is explaining complex evolutionary innovations that require the acquisition of multiple mutations. For metabolic innovations it has been shown that the addition of a single metabolic step in a pathway serves as a stepping stone towards the establishment of complex metabolic features in novel environments^14^. For millipedes, we have shown that independent lineages have gained similar levels of complexity independently, and the achievement of this complexity seems to have taken place in a step-wise manner. In the case of benzoquinones, the ability to produce phenols has been demonstrated to precede benzoquinone production and may have served as a stepping-stone for the production of hydroquinones, and these in place for the production of their corresponding benzoquinone. Even though these results are based on the most complete dataset to date, sampling is lower than ideal, considering the diversity of juliform lineages and chemistry. Nonetheless, the pattern that certain juliform families produce a considerably higher amount of chemicals than others is clear, and results so far support a gradual increase in complexity. Our study has major implications in the understanding of biodiversity, as adaptive traits greatly affect how taxa diversify through time. In response to predation, arthropods evolved novel defenses that enabled their escape from predators. These novel defenses facilitate species diversification among members of a clade sharing the same mechanism. After some time, predators develop the ability to adapt to these chemical defenses, and filling up empty niches, which allows for predator diversification. These predator-prey systems may produce a step-wise progression from simple to complex in the evolution of defense mechanisms^54–56^. For millipedes, an arms race with predators may have catalyzed the development of a metabolic stepping-stone process of evolutionary innovation. These novel biochemical defense secretion mechanisms potentially served as key innovations, allowing rapid diversification of the Juliformia and Polydesmida, which comprise approximately 75% of all nominal millipede species diversity in only four of 12 orders.

### Materials and Methods

Transcriptomes were sequenced, and *de novo* assembled for 34 taxa across Diplopoda, then combined with 15 previously published transcriptomes (Appendix S1, Table S1.1). The resulting dataset was used to: (i) resolve the phylogenetic relationships between orders using 2,638 orthologs and divergence dates using fossil calibrations; (ii) investigate the origin and evolution of phenol and benzoquinone production; and (iii) determine the directionality of the evolution of complex chemical systems in the benzoquinone pathway. We identified shifts in the number of molecules produced where chemical complexity appears to have increased over time in some lineages and decreased in others. The composition of chemical defenses was determined through GC-MS analysis of secretions and existing chemical records.

### RNA Extraction, Sequencing and Data Assembly

Total RNA was extracted from flash frozen or RNAlater preserved specimens using the TRIzol total RNA extraction method, purified with the RNeasy mini kit (Qiagen) and sequenced in-house at the Auburn University Core Genetics and Sequencing Laboratory using an Illumina Hi-Seq. 2500. Sequencing results (i.e. 100 bp paired end reads for each newly sequenced transcriptome) and fastq files from NCBI were *de novo* assembled in Trinity. New transcriptomes have been deposited into the NCBI Sequence Read Archive (SRA) (Appendix S1, Table S1.1). Resulting assemblies were analyzed in TransDecoder to produce predicted peptide sequences^57^.

### Orthology Assessment

A custom core ortholog set was generated to infer orthologs in HaMStR v13^58^. The set was produced using transcriptomes from eight millipedes (*Floridobolus sp*., *Sigmoria latior munda, Onomeris sp., Platydesmus sp., Polyxenidae sp., Pyrgodesmidae sp., Scoterpes sp*.) and one centipede (*Scutigera coleoptrata)*, which were chosen to cover a wide taxonomic sample. An all-versus-all BLASTP^59^ comparison was performed on the nine transcriptomes, with an e-value cut-off of 10^−5^, and used as input to perform Markov clustering in OrthoMCL 2.0^60^ with an inflation parameter of 2.1. The putatively orthologous groups (OGs) produced were processed through the following steps: (1) remove sequences shorter than 100 amino acids in length; (2) align each candidate OG with MAFFT^61^ using the automatic alignment strategy and a maxiterate value of 1,000; (3) produce an “approximately maximum likelihood tree” for each OG with FastTree 2^62^ using the “slow” and “gamma” options, by improving an initial neighbor-joining tree through minimum evolution with nearest neighbor interchange (NNI) subtree rearrangement, minimum evolution with subtree pruning regrafting (SPR) and maximum likelihood with NNI; (4) screen for paralogy using a tree-based approach in PhyloTreePruner^63^ by collapsing nodes with support values below 0.95, selecting a maximally inclusive tree (all taxa represented by no more than one sequence or more than one sequence forming a monophyletic group) and deleting putative paralogs (sequences falling outside of this maximally inclusive subtree) from the input alignment, where the longest sequence was retained for taxa with multiple sequences in a monophyletic group; and (5) discard all OGs with less than six of the nine taxa and/or not sampled for *Sigmoria latior munda*. These steps were performed using a modified version of the pipeline employed by Kocot *et al*.^64^. The pHMMs to be used in HaMStR were produced with hmmbuild and hmmcalibrate from the HMMER package^65^. Open reading frames produced by TransDecoder were searched against the 4,376 Millipede profile hidden Markov models (pHMMs), with *Sigmoria latior munda —*sampled in all OGs*—* as reference species. The steps outlined above were used to process the resulting putative OGs (modified from the Kocot *et al*.^64^ pipeline) and a supermatrix was produced (M1) with 2,638 orthologous sequence sets from 49 taxa by concatenation in FASconCAT^66^. Three reduced datasets were produced with 27% missing data (1250 orthologous sequence sets; M2), 21% missing data (625 orthologous sequence sets; M3), and 17% missing data (312 orthologous sequence sets; M4). We also calculated data informativeness for all partitions in PhyDesign^67^ and created two datasets with an informativeness score greater than 50 (M5) and the 312 most informative partitions (M6) for 30–200 Ma, focusing on resolving the position of Stemmiulida accurately (Appendix S1, Table S1.3).

### Phylogenetic Analyses

For all matrices (M1-M6) an optimal maximum likelihood tree was obtained in the program ExaML version 3.0.1^68^. Models of amino acid substitution were selected using the AUTOF command in ExaML. Node supports were estimated with 300 replicates generated and analyzed with the program RAxML and used to construct a majority-rule bootstrap consensus tree. A custom bash script (https://github.com/juanitarodrigueza/phylogenomics-pipeline/blob/master/bash_script) was used to automate the process and execute analyses on a high-performance computing (HPC) cluster. All analyses were conducted on the Auburn University CASIC HPS and Atrax (Bond Lab, Auburn University).

The program ASTRAL (Accurate Species TRee ALgorithm^69^) was used to infer a species tree from a series of unrooted gene trees. The ASTRAL approach works efficiently for large datasets and has been proposed to be more robust to incomplete lineage sorting, or deep coalescence, than maximum likelihood analysis of concatenated matrices. We first constructed individual gene trees for all partitions contained within matrices M1-M4. Maximum likelihood gene trees were generated in RAxML based on 100 random addition sequence replicates followed by 500 bootstrap replicates. Subsequent species tree estimation was inferred using ASTRAL v4.7.6, from all individual unrooted gene trees (and bootstrap replicates), under the multi-species coalescent model.

A reduced dataset with complete partitions for all taxa (M7; 38 partitions, 28 taxa) was generated for divergence time estimation in the program BEAST v1.8.1^70^ under a uncorrelated lognormal relaxed clock model^71,72^. Two calibration schemes (C1, C2) were used for the analysis. For C1, the most recent common ancestor (MRCA) of Chilopoda was given a lognormal prior of (offset) 383 (μ = 2, SD = 0.5) based on the fossil *Devonobius delta*^73^. The MRCA of Diplopoda was given a uniform prior from 423 to 580 Ma. The lower bound was set based on the youngest age of the oldest diplopod fossil *Pneumodesmus newmani* deposits^1^ and the upper bound was set based on the oldest Ediacaran fossils, as a maximum hard bound for major arthropod clades^74^. The MRCA of Glomerida + Glomeridesmida was given a lognormal prior of (mean in real space) 33.9 Ma (SD = 2) based on the fossil *Glomeris denticulata*^75^. The MRCA of Julida was given a lognormal prior of (mean in real space) 33.9 (SD = 1) based on the fossil *Parajulus cockerelli*^76^. The MRCA of Nematophora (Callipodida + Chordeumatida) was given a uniform prior of lower 307 Ma upper 580 Ma. The lower bound is based on the earliest date of the deposit for the oldest fossil Nematophora genus *Hexecontasoma*^77^. The MRCA of the MRCA of Pyrgodesmidae was given a lognormal prior of (mean in real space) 13.65 based on the fossil *Psochodesmus crescentis*^78^. The model of protein evolution was determined for each partition using the perl script ProteinModelSelection.pl in RAxML. A template xml file was generated in BEAUti v.1.8.1^70^ with all parameters and priors and each partition was transformed into an xml file using BEASTGen_v1.0^70^. For each partition, four separate MCMC (Markov Chain Monte Carlo) searches were run until ESS values –assessed with LogAnalyser in BEAST- for all parameters were greater than 200. Tree files were combined for all runs in a single partition and 10,000 trees were randomly sampled. Trees from all partitions were combined using LogCombiner v1.7.5. A consensus tree was produced using TreeAnnotator v1.8.1. A custom bash script was used to process input files and run all analyses (https://github.com/juanitarodrigueza/phylogenomics-pipeline/blob/master/bash_script). For the second calibration method (C2) all the available fossil evidence was used to calculate the Penultimate Gap (the interval between the two oldest fossils in a clade; PenG) and the ghost lineage length (the difference between the oldest fossils for each of two sister lineages; GLin) as described by Norris *et al*. not in Reference section (http://biorxiv.org/content/biorxiv/early/2015/01/24/014340.full.pdf). These values were used to produce a prior distribution of the true age of crown-group Chilopoda and stem Helminthomorpha. The MRCA of crown-group Chilopoda was given a lognormal prior of (offset) 417 Ma (μ = 1.74, SD = 1,81). The MRCA of Helminthomorpha + Pentazonia was given a lognormal prior of (offset) 423 Ma (μ = 1.20,SD = 1.81). A chronogram containing all taxa was generated for ancestral character reconstruction using a penalized likelihood method using the chronos command in the R package ape^47^. The 95% highest posterior density dates obtained for the C2 BEAST analysis were incorporated as constraints for node ages.

### Chemical Analysis

To collect the defensive compounds, live specimens were placed in a glass vial with 0.5 mL 100% methanol for a full body extraction. One specimen was sampled for each taxon. To avoid contamination, the glass vials had caps lined with Teflon. The methanol was analyzed using a Shimadzu QP-5000 GC/MS or Shimadzu QP-2010 GC/MS equipped with an RTX-5, 30 m × 0.25 mm i.d. column. The instruments were programmed from 60 °C to 250 °C at 10 °/min and held at that temperature for 20 min. To determine compounds, we used published data on mass spectra of quinone isomers. We also used and authentic samples of quinones as well as o-cresol, m-cresol, and p-cresol, present in our analytical chemistry facility. Retention indices were not used.

### Ancestral Character Reconstruction

Chemical composition of defense secretion was mapped as a multistate character with states (1) No chemical, (2) Quinazolinone alkaloids, (3) Heterocyclic nitrogen-containing compounds, (4) Terpenes, (5) Benzoquinones and hydroquinones, (6) Phenol, (7) Cyanogenics. For the hypothetical phenol-benzoquinone pathway a single character was mapped with states (1) No chemical production, (2) Phenol production, (3) Benzoquinone production, and as a continuous character with states as number of chemical defense molecules found on hypothetical benzoquinone pathway^79^ (Fig. 2A), and assuming intermediate states as present (i.e. if a chemical found on the edge of the pathway was found, its hypothetical precursors were counted as present even if not reported in chemical results or literature) (see Supplemental Table S1.2, and S1.3). This character was scored at the family level, so that each terminal taxon represented the entire family lineage and its states corresponded to all chemical information available for the entire family. Discrete character states were mapped using a maximum likelihood approach implemented using the rayDISC command in the R package corHMM^80^. For chemical production, likelihood for the models of character evolution equal rates (ER), symmetrical (SYM) and all rates different (ARD) was calculated. The best model was determined using a likelihood ratio test. For phenol and benzoquinones we established a rate matrix that assumed different rates between transitions. This rate matrix assumed phenol as ancestral to benzoquinone as has been suggested for other arthropods^22^ and a single rate involved in losing the ability to produce one or all chemicals. The log-likelihood of this reconstruction was compared to that of other three models of character evolution: equal rates (ER), symmetrical (SYM) and all rates different (ARD). A likelihood-ratio test was performed to select among these varying models of character evolution. The model that best fit the data was the rate matrix model. The root state was determined using the procedure described by Maddison *et al*.^81^ and FitzJohn *et al*.^82^, treating the root state as a nuisance parameter and using an alternative root assignment that weights each root state according to its probability of giving rise to the extant data, given the model parameters and the tree. Continuous characters were mapped using the contMap command from the R package phytools^83^.

### Data availability

Transcriptomes are available at NCBI Sequence Read Archive (SRA), assembled transcriptomes, ortholog alignments, and matrices are available from Dryad Data Repository.

### Code availability

Scripts used in the analysis of these data are available at https://github.com/juanitarodrigueza.

## Electronic supplementary material

Supplementary Material
