# Supplementary material for: Step-wise evolution of complex chemical defenses in millipedes: a phylogenomic approach
Source: Sci Rep. 2018 Feb 16;8:3209. doi: 10.1038/s41598-018-19996-6 (PMC5816663; doi:10.1038/s41598-018-19996-6)
Supplement: Supplementary file 1 — Supplementary Material [file 41598_2018_19996_MOESM1_ESM.doc]

Step-wise evolution of complex chemical defenses in millipedes: a phylogenomic approach

Juanita Rodriguez ^a,b^, Tappey H. Jones ^c^, Petra Sierwald ^d^, Paul E. Marek ^e^, William A. Shear ^f^, Michael S. Brewer ^g^, Kevin M. Kocot ^h^, and Jason E. Bond ^a*^

a Department of Biological Sciences, Auburn University, Auburn, AL, 36849, USA

b CSIRO, Australian National Insect Collection, Canberra, ACT, 2601, Australia

c Department of Chemistry, Virginia Military Institute, Lexington, VA

24450, USA

d Zoology Department, The Field Museum, Chicago, IL 60605, USA

e Department of Entomology, Virginia Tech, Blacksburg, VA 24061, USA

f Biology Department, Hampden-Sydney College, Farmville, VA 23943, USA

g Department of Biology, East Carolina University, Greenville, NC, 27858, USA

h Department of Biological Sciences, University of Alabama, Tuscaloosa, AL 35487,USA

*Corresponding author

Email: jeb0037@auburn.edu

**Table S1.1: Transcriptome and genome data sequenced and analyzed. Voucher specimen numbers in the Auburn University Museum of Natural History (AUM) and Sequence Read Archive (SRA) accession numbers are shown. Asterisks indicate taxa excluded from all except penalized likelihood dating.**

| **Class** | **Order** | **Family** | **Species** | **Voucher ID** | **NCBI SRA** | **# contigs** | **# partitions** | **Reference** |
| --- | --- | --- | --- | --- | --- | --- | --- | --- |
| Diplopoda | Callipodida | Abacion | *Abacion_sp* | MITS007 |  | 46986 | 1533 | This study |
|  |  | Abacionidae | *Abacion_magnum* |  |  | 15681 | 803 | (1) |
|  | Chordeumatida | Striariidae | *Striaria_sp* | MITS013 |  | 53961 | 1774 | This study |
|  |  | Trichopetalidae | *Scoterpes_sp* | AUMS15047 |  | 121879 | 2003 | This study |
|  |  | Cleidogonidae | *Cleidogona_sp* |  |  | 16572 | 850 | (1) |
|  | Glomerida | Glomeridae | *Onomeris_sp* |  |  | 71678 | 1917 | (1) |
|  | Glomeridesmida | Glomeridesmidae | *Glomeridesmus_sp* | MITS489 |  | 67130 | 1964 | This study |
|  |  |  | *Glomeridesmus_sp* |  |  | 20181 | 1453 | (1) |
|  | Julida | Paeromopodidae | *Californiulus_sp* | MITS203 |  | 111379 | 2103 | This study |
|  |  | Nemasomatidae | *Orinisobates_nigrior* | MITS622 |  | 87212 | 2044 | This study |
|  |  | Parajulidae | *Uroblaniulus_sp* | MITS883 |  | 78216 | 2120 | This study |
|  |  | Julidae* |  | MITS682 |  | 18251 | 26 | This study |
|  | Platydesmida | Andrognathidae | *Brachycybe_lecontii* |  |  | 18570 | 1646 | (1) |
|  |  |  | *Andrognathus_corticarius* | MITS652 |  | 78205 | 1999 | This study |
|  |  | Platydesmidae | *Platydesmus_sp* | MITS438 |  | 41950 | 1848 | This study |
|  | Polydesmida | Chelodesmidae | *Chondromorpha_cairoensis* | MITS434 |  | 32890 | 510 | This study |
|  |  |  | *Trichomorpha_sp* | MITS457 |  | 30706 | 814 | This study |
|  |  | Cyrtodesmidae | *Cyrtodesmus_sp* | MITS521 |  | 41429 | 1982 | This study |
|  |  | Paradoxomatidae | *Asiomorpha_coarctata* | MITS500 |  | 70971 | 1877 | This study |
|  |  | Polydesmidae | *Polydesmus_angustus* |  | SRR1047642 | 13692 | 800 | NCBI |
|  |  |  | *Pseudopolydesmus_sp* |  |  | 18876 | 1687 | (1) |
|  |  | Paradoxomatidae | *Mestosoma_sp* | MITS539 |  | 17938 | 1264 | This study |
|  |  | Pyrgodesmidae | *Pyrgodesmidae_sp* | MITS018 |  | 65763 | 2206 | This study |
|  |  | Sphaeriodesmidae | *Desmonus_earlei* | MITS016 |  | 46902 | 2148 | This study |
|  |  | Xystodesmidae | *Xystodesmidae_sp* | AUMS5334 |  | 19560 | 937 | This study |
|  |  | Xystodesmidae | *Thrinaxoria_sp* | M1 |  | 24290 | 1468 | This study |
|  |  | Xystodesmidae | *Sigmoria_latior_munda* | MITS006 |  | 62565 | 1479 | This study |
|  |  | Xystodesmidae | *Cleptoria_abbotti* | AUMS15706 |  | 91075 | 1542 | This study |
|  | Polyxenida | Polyxenidae | *Polyxenus_lagurus* |  | SRR1048056 | 10008 | 192 | NCBI |
|  |  |  | *Polyxenidae_sp* | 2438-JEB-0005 |  | 27343 | 1242 | This study |
|  | Polyzoniida | Polyzoniidae | *Petaserpes_sp* |  |  | 12154 | 959 | (1) |
|  |  | Siphonotidae | *Rhinotus_purpureus* | MITS524 |  | 50595 | 1844 | This study |
|  | Siphonophorida | Siphonophoridae | *Siphonophorus_sp* | MITS482 |  | 43856 | 213 | This study |
|  |  |  | *Siphonophorus_sp* | MITS557 |  | 42435 | 1839 | This study |
|  | Sphaerotheriida | Sphaerotheriidae | *Sphaerotheriidae_sp* | AUMS15748 |  | 67616 | 1900 | This study |
|  | Spirobolida | Floridobolidae | *Floridobolus_sp* | AUMS12965 |  | 52015 | 1645 | This study |
|  |  | Rhinocricidae | *Anadenobolus_sp* | AUMS15384 |  | 73070 | 2082 | This study |
|  |  | Spirobolidae | *Narceus_sp* | AUMS5195 |  | 22709 | 1280 | This study |
|  |  |  | *Tylobolus* | AUMS15746 |  | 94628 | 1938 | This study |
|  |  | Spirobollelidae | *Microspirobolus_sp* | MITS504 |  | 33140 | 1364 | This study |
|  | Spirostreptida | Cambalidae | *Cambala_annulata* |  |  | 14071 | 1234 | (1) |
|  |  | Spirostreptidae | *Orthoporus_sp* | MITS483 |  | 46470 | 2892 | This study |
|  |  |  | *Orthoporus_sp* | MITS431 |  | 43621 | 1592 | This study |
|  | Stemmiulida | Stemmiulidae | *Prostemmiulus_sp* |  |  | 10524 | 664 | (1) |
|  |  |  | *Prostemmiulus_loomisi* | MITS464 |  | 55359 | 840 | This study |
| Symphyla | Symphyla | Scolopendrellidae | *Symphylella_vulgaris* |  | SRR768329 | 34865 | 793 | NCBI |
| Chilopoda | Lithobiomorpha | Lithobiidae | *Lithobius_sp* |  |  | 33692 | 1258 | (1) |
|  | Scolopendromorpha | Cryptopidae | *Cryptops_hortensis* |  | SRR1153457 | 135118 | 1686 | NCBI |
|  | Scutigeromorpha | Scutigeridae | *Scutigera coleoptrata* |  | SRX462011 | 211777 | 1617 | NCBI |

**Table S1.2. Chemical composition of defense secretion for families included in this analysis. Relative amounts, source of chemical information and AUM voucher number are indicated.**

| **Order** | **Family** | **Genus** | **Chemical** | **Relative Amount** | **Voucher ID** | **Ref.** |
| --- | --- | --- | --- | --- | --- | --- |
| Julida | Julidae | *Allajulus* | (E)-Alkenals |  |  | (2) |
|  |  |  | 2-hydroxy-3-methyl-1,4 benzoquinone | 2.7 |  |  |
|  |  |  | 2-methoxy-3-methyl-1,4-benzoquinone | 44.3 |  |  |
|  |  |  | 2,3-dimethoxy-1,4-benzoquinone | 2.1 |  |  |
|  |  |  | 2,3-dimethoxy-5-methyl-1,4-benzoquinone | 0.7 |  |  |
|  |  | *Brachyulus* | 1,4-benzoquinone | trace |  | (3) |
|  |  |  | 2-hydroxy-3-methyl-1,4 benzoquinone | 0.5 |  |  |
|  |  |  | 2-methoxy-3-methyl-1,4-benzoquinone | 20.9 |  |  |
|  |  |  | 2-methoxy-6-methyl-1,4 benzoquinone | 1.9 |  |  |
|  |  |  | 2-methyl-1,4-benzoquinone | 54.3 |  |  |
|  |  |  | 2-methyl-hydroquinone | 0.1 |  |  |
|  |  |  | 2,3-dimethoxy-5-methyl-1,4-benzoquinone | 3.9 |  |  |
|  |  |  | 2,3-dimethoxy-5-methyl-hydroquinone | 0.3 |  |  |
|  |  |  | 3-hydroxy-5-methoxy-2-methyl-1,4-benzoquinone | 0.3 |  |  |
|  |  |  | dimethoxy-hydroxy-benzoquinone isomers | trace |  |  |
|  |  |  | p-cresol | 21.4 |  |  |
|  |  |  | phenol | trace |  |  |
|  |  | *Cylindroiulus* | 1,4-benzoquinone | 0.2 |  | (3) |
|  |  |  | 2-hydroxy-3-methyl-1,4 benzoquinone | 0.5 |  |  |
|  |  |  | 2-methoxy-3-methyl-1,4-benzoquinone | 20.9 |  |  |
|  |  |  | 2-methoxy-6-methyl-1,4 benzoquinone | 0.5 |  |  |
|  |  |  | 2-methyl-1,4-benzoquinone | 25.2 |  |  |
|  |  |  | 2-methyl-hydroquinone | 0.2 |  |  |
|  |  |  | 2,3-dimethoxy-1,4-benzoquinone | 0.5 |  |  |
|  |  |  | 2,3-dimethoxy-5-methyl-1,4-benzoquinone | 1.1 |  |  |
|  |  |  | p-cresol | 50.6 |  |  |
|  |  |  | phenol | 0.1 |  |  |
|  |  | *Lamellotyphlus* | 2-methoxy-5-methyl-1,4-benzoquinone | 2.0 |  | (4) |
|  |  |  | 2-methoxy-6-methyl-1,4-benzoquinone | 0.4 |  |  |
|  |  |  | 2,3-dimethoxy-5-methyl-1,4-benzoquinone | 86.7 |  |  |
|  |  |  | dimethoxy-methylhydroquinone/isomer1 | 0.5 |  |  |
|  |  |  | 2,6-dimethoxy-3-methyl-1,4-benzoquinone | 10.0 |  |  |
|  |  |  | methoxy-methylhydroquinoneisomer2 | 0.3 |  |  |
|  |  | *Serboiulus* | 2-methyl-1,4-benzoquinone | 18.8 |  | (4) |
|  |  |  | 4-methylphenol | 0.4 |  |  |
|  |  |  | 2-ethyl-1,4-benzoquinone | 56.7 |  |  |
|  |  |  | 4-ethylphenol | 0.1 |  |  |
|  |  |  | 2-methoxy-3-methyl-1,4-benzoquinone | 14.4 |  |  |
|  |  |  | 2-ethyl-3-methoxy-1,4-benzoquinone | 2.4 |  |  |
|  |  |  | 2,3-dimethoxy-1,4-benzoquinone | 0.9 |  |  |
|  |  |  | 2-methylhydroquinone | 0.2 |  |  |
|  |  |  | 2,3-dimethoxyhydroquinone | 0.6 |  |  |
|  |  |  | 2-methyl-3,4-methylenedioxyphenole | 1.0 |  |  |
|  |  |  | 2,3-dimethoxy-5-methyl-1,4-benzoquinone | 0.2 |  |  |
|  |  |  | 2-ethyl-hydroquinone | 2.7 |  |  |
|  |  |  | dimethoxy-methylhydroquinone/isomer1 | 0.8 |  |  |
|  |  |  | 2,3-dimethoxy-5,6-dimethylhydroquinone | 0.6 |  |  |
|  |  | *Serboiulus* | 2-methyl-1,4-benzoquinone | 25.0 |  | (4) |
|  |  |  | 2-ethyl-1,4-benzoquinone | 48.5 |  |  |
|  |  |  | 2-hydroxy-3-methyl-1,4-benzoquinone | 2.0 |  |  |
|  |  |  | 2-methoxy-3-methyl-1,4-benzoquinone | 7.0 |  |  |
|  |  |  | 2-ethyl-3-methoxy-1,4-benzoquinone | 0.8 |  |  |
|  |  |  | 2-methoxy-1,4-benzoquinone | 0.6 |  |  |
|  |  |  | 2,3-dimethoxy-1,4-benzoquinone | 4.0 |  |  |
|  |  |  | 2-methylhydroquinone | 0.2 |  |  |
|  |  |  | 2-methoxy-5-methyl-1,4-benzoquinone | 0.1 |  |  |
|  |  |  | 2,3-dimethoxyhydroquinone | 1.3 |  |  |
|  |  |  | 2-methyl-3,4-methylenedioxyphenole | 4.0 |  |  |
|  |  |  | 2,3-dimethoxy-5-methyl-1,4-benzoquinone | 1.3 |  |  |
|  |  |  | 2-ethyl-hydroquinone | 0.9 |  |  |
|  |  |  | dimethoxy-methylhydroquinone/isomer1 | 0.6 |  |  |
|  |  | *Serboiulus* | 2-methyl-1,4-benzoquinone | 14.6 |  | (4) |
|  |  |  | 2-ethyl-1,4-benzoquinone | 66.3 |  |  |
|  |  |  | 2-hydroxy-3-methyl-1,4-benzoquinone | 0.8 |  |  |
|  |  |  | 2-methoxy-3-methyl-1,4-benzoquinone | 1.4 |  |  |
|  |  |  | 2-ethyl-3-methoxy-1,4-benzoquinone | 0.1 |  |  |
|  |  |  | 2,3-dimethoxy-1,4-benzoquinone | 1.0 |  |  |
|  |  |  | 2-methylhydroquinone | 0.1 |  |  |
|  |  |  | 2,3-dimethoxyhydroquinone | 0.4 |  |  |
|  |  |  | 2-methyl-3,4-methylenedioxyphenole | 1.2 |  |  |
|  |  |  | 2,3-dimethoxy-5-methyl-1,4-benzoquinone | 10.7 |  |  |
|  |  |  | dimethoxy-methylhydroquinone/isomer1 | 0.1 |  |  |
|  |  | *Styrioiulus* | 1,4-benzoquinone | 0.1 |  | (3) |
|  |  |  | 2-methyl-1,4-benzoquinone | 3.5 |  |  |
|  |  |  | p-cresol | 93.1 |  |  |
|  |  |  | phenol | 1.3 |  |  |
|  |  | *Typhloiulus* | 1,4-benzoquinone | 0.1 |  | (3) |
|  |  | *Typhloiulus* | 2-hydroxy-3-methoxy-1,4-benzoquinone | 0.2 |  | (3) |
|  |  |  | 2-hydroxy-3-methyl-1,4-benzoquinone | 0.4 |  |  |
|  |  |  | 2-methoxy-3-methyl-1,4-benzoquinone | 8.1 |  |  |
|  |  |  | 2-methoxy-5-methyl-1,4-benzoquinone | 0.7 |  |  |
|  |  |  | 2-methoxy-6-methyl-1,4 benzoquinone | 0.6 |  |  |
|  |  |  | 2-methyl-1,4-benzoquinone | 31.8 |  |  |
|  |  |  | 2,3-dimethoxy-1,4-benzoquinone | trace |  |  |
|  |  |  | 3-hydroxy-5-methoxy-2-methyl-1,4-benzoquinone | 5.4 |  |  |
|  |  |  | dimethoxy-hydroxy-benzoquinone isomers | 0,.7-13.6 |  |  |
|  |  |  | p-cresol | 31.8 |  |  |
|  |  |  | phenol | 0.7 |  |  |
|  |  | *Typhloiulus* | 1,4-benzoquinone | 0.9 |  | (4) |
|  |  |  | 2-methyl-1,4 –benzoquinone | 35.2 |  |  |
|  |  |  | 2-ethyl-1,4-benzoquinone | 34.9 |  |  |
|  |  |  | 2-hydroxy-3-methyl-1,4-benzoquinone | 3.5 |  |  |
|  |  |  | 2-methoxy-3-methyl-1,4-benzoquinone | 21.1 |  |  |
|  |  |  | 2-ethyl-3-methoxy-1,4-benzoquinone | 1.3 |  |  |
|  |  |  | 2,3-dimethoxy-1,4-benzoquinone | 2.7 |  |  |
|  |  |  | 2-methylhydroquinone | 0.2 |  |  |
|  |  |  | 2,3-dimethoxyhydroquinone | 0.1 |  |  |
|  |  | *Typhloiulus* | 1,4-benzoquinone | 1.9 |  | (4) |
|  |  |  | 2-methyl-1,4-benzoquinone | 25.7 |  |  |
|  |  |  | 2-ethyl-1,4-benzoquinone | 47.9 |  |  |
|  |  |  | 2-hydroxy-3-methyl-1,4-benzoquinone | 0.5 |  |  |
|  |  |  | 2-methoxy-3-methyl-1,4-benzoquinone | 20.2 |  |  |
|  |  |  | 2-ethyl-3-methoxy-1,4-benzoquinone | 2.3 |  |  |
|  |  |  | 2,3-dimethoxy-1,4-benzoquinone | 1.4 |  |  |
|  |  | *Typhloiulus* | 2-methyl-1,4-benzoquinone | 0.6 |  | (4) |
|  |  |  | 2-hydroxy-3-methyl-1,4-benzoquinone | 0.7 |  |  |
|  |  |  | 2-methoxy-3-methyl-1,4-benzoquinone | 81.9 |  |  |
|  |  |  | 2-ethyl-3-methoxy-1,4-benzoquinone | 0.5 |  |  |
|  |  |  | 2-methoxy-1,4-benzoquinone | 0.7 |  |  |
|  |  |  | 2,3-dimethoxy-1,4-benzoquinone | 6.0 |  |  |
|  |  |  | 2-methoxy-5-methyl-1,4-benzoquinone | 0.3 |  |  |
|  |  |  | 2,3-dimethoxyhydroquinone | 1.5 |  |  |
|  |  |  | 2-methyl-3,4-methylenedioxyphenole | 3.6 |  |  |
|  |  |  | 2,3-dimethoxy-5-methyl-1,4-benzoquinone | 2.0 |  |  |
|  |  |  | dimethoxy-methylhydroquinone/isomer1 | 0.8 |  |  |
|  |  |  | methylparaben | 1.1 |  |  |
|  |  | *Typhloiulus* | 2-methyl-1,4-benzoquinone | trace |  | (4) |
|  |  |  | 2-hydroxy-3-methyl-1,4-benzoquinone | 5.6 |  |  |
|  |  |  | 2-methoxy-3-methyl-1,4-benzoquinone | 74.4 |  |  |
|  |  |  | 2,3-dimethoxy-1,4-benzoquinone | 3.2 |  |  |
|  |  |  | 2,3-dimethoxyhydroquinone | 2.7 |  |  |
|  |  |  | 2-methyl-3,4-methylenedioxyphenole | 13.2 |  |  |
|  |  |  | 2,3-dimethoxy-5-methyl-1,4-benzoquinone | 0.3 |  |  |
|  |  |  | dimethoxy-methylhydroquinone/isomer1 | 0.5 |  |  |
|  |  | *Typhloiulus* | 2-methyl-1,4-benzoquinone | 11.4 |  | (4) |
|  |  |  | 2-hydroxy-3-methyl-1,4-benzoquinone | 8.1 |  |  |
|  |  |  | 2-ethyl-1,4-benzoquinone | 22.7 |  |  |
|  |  |  | 2-methoxy-3-methyl-1,4-benzoquinone | 6.7 |  |  |
|  |  |  | 2,3-dimethoxy-1,4-benzoquinone | 15.9 |  |  |
|  |  |  | 2-ethyl-3-methoxy-1,4-benzoquinone | 3.0 |  |  |
|  |  |  | 2,3-dimethoxyhydroquinone | 9.9 |  |  |
|  |  |  | 2,3-dimethoxy-1,4-benzoquinone | 15.9 |  |  |
|  |  |  | 2-methyl-3,4-methylenedioxyphenole | 18.5 |  |  |
|  |  |  | 2,3-dimethoxy-5-methyl-1,4-benzoquinone | 3.8 |  |  |
|  |  | *Typhloiulus* | 2-methyl-1,4-benzoquinone | 0.1 |  | (4) |
|  |  |  | 2-ethyl-1,4-benzoquinone | 0.3 |  |  |
|  |  | *Typhloiulus* | 2-methyl-1,4-benzoquinone | 13.1 |  | (4) |
|  |  |  | 4-methylphenol | 0.1 |  |  |
|  |  |  | 2-ethyl-1,4-benzoquinone | 35.7 |  |  |
|  |  |  | 2-hydroxy-3-methyl-1,4-benzoquinone | 6.9 |  |  |
|  |  |  | 4-ethylphenol | 0.1 |  |  |
|  |  |  | 2-methoxy-3-methyl-1,4-benzoquinone | 13.2 |  |  |
|  |  |  | 2-ethyl-3-methoxy-1,4-benzoquinone | 1.8 |  |  |
|  |  |  | 2,3-dimethoxy-1,4-benzoquinone | 2.1 |  |  |
|  |  |  | 2-methylhydroquinone | 0.8 |  |  |
|  |  |  | 2,3-dimethoxyhydroquinone | 4.2 |  |  |
|  |  |  | 2-methyl-3,4-methylenedioxyphenole | 15.5 |  |  |
|  |  |  | 2-ethyl-hydroquinone | 4.0 |  |  |
|  |  |  | 2,3,5,6-tetramethylhydroquinone | 1.8 |  |  |
|  |  | *Typhloiulini* n.sp. | 1,4-benzoquinone | 0.1 |  | (4) |
|  |  |  | phenol | 0.7 |  |  |
|  |  |  | 2-methyl-1,4-benzoquinone | 31.8 |  |  |
|  |  |  | 4-methylphenol | 31.8 |  |  |
|  |  |  | 2-hydroxy-3-methyl-1,4-benzoquinone | 0.4 |  |  |
|  |  |  | 2-methoxy-3-methyl-1,4-benzoquinone | 8.1 |  |  |
|  |  |  | 2,3-dimethoxy-1,4-benzoquinone | trace |  |  |
|  |  |  | 2-methoxy-5-methyl-1,4-benzoquinone | 0.7 |  |  |
|  |  |  | 2-methoxy-6-methyl-1,4-benzoquinone | 0.6 |  |  |
|  |  |  | 2-hydroxy-3-methoxy-1,4-benzoquinone | 0.2 |  |  |
|  |  |  | 2,3-dimethoxy-5-methyl-1,4-benzoquinone | 1.3 |  |  |
|  |  |  | methylparaben | 0.8 |  |  |
|  |  |  | dimethoxy-hydroxy-benzoquinone isomer | 0.2 |  |  |
|  |  |  | 2-hydroxy-3-methoxy-5-methyl-1,4-benzoquinone | 5.4 |  |  |
|  |  |  | dimethoxy-hydroxy-methyl-benzoquinoneisomer1 | 13.6 |  |  |
|  |  |  | dimethoxy-hydroxy-methyl-benzoquinoneisomer2 | 1.8 |  |  |
|  |  |  | dimethoxy-hydroxy-methyl-benzoquinoneisomer3 | 0.7 |  |  |
|  |  | *Unciger* | 1,4-benzoquinone | 1.3 |  | (5) |
|  |  |  | 2-ethyl-1,4-benzoquinone | 0.1 |  |  |
|  |  |  | 2-hydroxy-3-methyl-1,4-benzoquinone | 3.2 |  |  |
|  |  |  | 2-methoxy-1,4-benzoquinone | 0.2 |  |  |
|  |  |  | 2-methoxy-3-methyl-1,4-benzoquinone | 26.1 |  |  |
|  |  |  | 2-methyl-1,4-benzoquinone | 56.2 |  |  |
|  |  |  | 2-methyl-hydroquinone | 1 |  |  |
|  |  |  | 2,3-dimethoxy-1,4-benzoquinone | 0.2 |  |  |
|  |  |  | 2,3-dimethoxy-5-methyl-1,4-benzoquinone | 0.1 |  |  |
|  |  |  | 2,3-dimethoxy-5-methyl-hydroquinone | 1.2 |  |  |
|  |  |  | 2,3-dimethoxy-hydroquinone | 0.9 |  |  |
|  |  |  | hydroquinone | 0.4 |  |  |
|  |  |  | p-cresol | trace |  |  |
|  |  |  | phenol | 0.1 |  |  |
|  | Nemasomatidae | *Orinisobates* | 2-methyl-1,4-benzoquinone | 26 | 622 | This study |
|  |  |  | 2- methyl-1,4 hydroquinone | 15 |  |  |
|  | Paeromopodidae | *Californiulus* | FAMES |  |  | This study |
|  | Parajulidae | *Oriulus* | 2-methoxy-3-methyl-1,4-benzoquinone; o-cresol | no info |  | (6) |
|  |  | *Uroblaniulus* | 1,4-benzoquinone | no info |  | This study |
|  |  |  | 2,3-dimethoxy-1,4-benzoquinone | no info |  |  |
| Spirobolida | Floridobolidae | *Floridobolus* | 2-hydroxy-3-methyl-1,4-benzoquinone | 0.1-0.2 |  | (7) |
|  |  |  | 2-methoxy-3-methyl-1,4-benzoquinone | 37-46 |  | (8) |
|  |  |  | 2-methyl-1,4-benzoquinone | 46-61 |  |  |
|  |  |  | 2,3-dimethoxy-1,4-benzoquinone | 1.3-5.3 |  | (9) |
|  |  |  | 2,3-dimethoxy-5-methyl-1,4-benzoquinone | 0.2-1.5 |  |  |
|  |  |  | 2,5-dimethyl-3-methoxy-1,4-benzoquinone | 0.2-0.5 |  |  |
| Spirobolida | Rhinocricidae | *Acladocricus* | 1,4-benzoquinone | 1.1 |  |  |
|  |  |  | 2-3-dimethoxy-1,4-benzoquinone | 0.9 |  |  |
|  |  |  | 2-ethyl-1,4-benzoquinone | 10.7 |  |  |
|  |  | *Acladocricus* | 2-hydroxy-3-methyl-1,4-benzoquinone | 88.1 |  | (10) |
|  |  |  | 2-methoxy-3-methyl-1,4-benzoquinone | 8.7 |  |  |
|  |  |  | 2-methoxy-3-methyl-1,4-benzoquinone | large amount |  |  |
|  |  |  | 2-methoxy-3-methylhydroquionone | 1.8 |  | (10) |
|  |  |  | 2-methoxy-3,6-dimethyl-1,4-benzoquinone | 100 |  | (11) |
|  |  | *Acladocricus* | 2-methyl-1,4-benzoquinone | 21.3 |  | (10) |
|  |  |  | 2-methyl-1,4-benzoquinone | large amount |  |  |
|  |  |  | 2-methyl-3,4-methylenedioxyphenol | 3.4 |  |  |
|  |  |  | 2-methyl-hydroquinone | trace |  | (11) |
|  |  |  | 2,3-dimethoxy-5-methyl-1,4-benzoquinone | 0.5 |  | (10, 12) |
|  |  |  | 2,3-dimethoxy-5-methyl-hydroquinone | 2.7 |  | (10) |
|  |  |  | 2,3-dimethoxy-hydroquinone | 5.8 |  |  |
|  |  |  | hydroquinone | large amount |  |  |
|  |  | *Anadenobolus* | 2-methoxy-3-methyl-1,4-benzoquinone |  |  | (13) |
|  |  |  | 2-methoxy-3-methylhydroquinone | large amount |  |  |
|  |  |  | 2-methyl-1,4-benzoquinone |  |  |  |
|  |  |  | 2-methylhydroquinone | 4.65 |  |  |
|  |  | *Orthoporus* | 2,6-dimethoxy-3-methyl-1,4-benzoquinone | 18 | 487 | This study |
|  |  |  | 2,6-dimethoxy-3-methyl-1,4-benzoquinone | 4 |  |  |
|  |  |  | 3,4,5-trimethoxyphenol |  |  |  |
|  |  |  | 2-methoxy-3-methyl-1,4-benzoquinone | 9.65 |  |  |
|  |  |  | 2-methoxy-3-methylhydroquinone | 4.6 | 468 | This study |
|  |  |  | 2-methoxy-5-methyl-1,4-benzoquinone | 2.74 |  |  |
|  |  |  | 2-methoxy-6-methyl-1,4 benzoquinone | 53.75 |  |  |
|  |  |  | 2-methyl-hydroquinone | 9.6 |  |  |
|  |  |  | 2,3-dimethoxy-5-methyl-1,4-benzoquinone | 18.03 |  |  |
|  |  |  | 2,5-dimethoxy-3-methyl-hydroquinone | 2 |  |  |
|  |  |  | 2,6-dimethoxy-3-methyl-1,4-benzoquinone | 7.12 |  |  |
|  |  |  | 2,6-dimethoxy-3-methyl-1,4-benzoquinone | 18 |  |  |
|  |  |  | x | 54 |  |  |
|  |  |  | 3,4,5-trimethoxyphenol |  |  |  |
|  | Spirobolidae | *Narceus* | 2-methoxy-3-methyl-1,4-benzoquinone | 8.5 | 469 | This study |
|  |  |  | 2-methyl-hydroquinone | 4.46 | 320 | This study |
|  |  |  | 2-methoxy-3-methyl-1,4-benzoquinone | no info |  | (14) |
|  |  |  | 2-methyl-1,4-benzoquinone | no info |  |  |
|  |  | *Spirobolellus* | 2-methoxy-3-methyl-1,4-benzoquinone | 95 |  | (15) |
|  |  |  | 2-methoxy-3-methylhydroquinone | trace |  |  |
|  |  |  | 2,3-dimethoxy-1,4-benzoquinone | 5 |  |  |
|  |  |  | 2,3-dimethoxy-5-methyl-1,4-benzoquinone | trace |  |  |
|  |  |  | 2,3-dimethoxy-hydroquinone | trace |  |  |
|  | Spirobollelidae? | *Microspirobolus?* | 2,3-dimethoxy-5-methyl-1,4-benzoquinone | 10.35 | 506 | This study |
|  |  |  | 2,6-dimethoxy-3-methyl-1,4-benzoquinone | 3.34 |  |  |
|  |  |  | 2-methoxy-3-methyl-1,4-benzoquinone | 5.48 |  |  |
|  | Spirobollelidae? | *Microspirobolus?* | 2-methoxy-6-methyl-1,4 benzoquinone | 5.95 | 507 | This study |
|  |  |  | 2-methoxy-6-methyl-hydroquinone | 27.35 |  |  |
|  |  |  | 2-methyl-hydroquinone | 71.4 |  |  |
|  |  |  | 2,3-dimethoxy-5-methyl-1,4-benzoquinone | 28.6 |  |  |
|  |  |  | 2,6-dimethoxy-3-methyl-1,4-benzoquinone | 7.79 |  |  |
|  |  |  | 2,6-dimethoxy-3-methyl-1,4-benzoquinone | 3.44 |  |  |
|  |  |  | 3,4-methoxyphenol |  |  |  |
|  |  |  | 3,4,5-trimethoxyphenol? Not in amounts data |  |  |  |
| Spirostreptida | Cambalidae | *Cambala* | 2-methoxy-3-methyl-1,4-benzoquinone |  |  | (16) |
|  |  |  | |  |  |  |  | | --- | --- | --- | --- | |  | 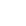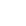 |  | 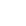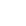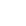 |  | 2-methyl-1,4-benzoquinone | | --- | |  |  |  |
|  | Spirostreptidae | *Telodeinopus* | 1,4-benzoquinone | 0.09 |  | (17) |
|  |  |  | 2-ethyl-1,4-benzoquinone | 0.04 |  |  |
|  |  |  | 2-methoxy-1,4-benzoquinone | 0.13 |  |  |
|  |  |  | 2-methoxy-3-methyl-1,4-benzoquinone | 69.06 |  |  |
|  |  |  | 2-methoxy-3-methylhydroquinone | trace |  |  |
|  |  |  | 2-methoxy-hydroquinone | nd |  |  |
|  |  |  | 2-methyl-1,4-benzoquinone | 21.5 |  |  |
|  |  |  | 2-methyl-hydroquinone | 1.69 |  |  |
|  |  |  | 2,3-dimethoxy-1,4-benzoquinone | 4.23 |  |  |
|  |  |  | 2,3-dimethoxy-5-methyl-1,4-benzoquinone | 0.26 |  |  |
|  |  |  | 2,3-dimethoxy-hydroquinone | n.d. |  |  |
|  |  |  | Hydroquinone | 0.1 |  |  |
|  |  |  | Naphthoquinone | 0.03 |  |  |
| Stemmiulida | Stemmiulidae | *Prostemmiulus* | Benzyl alcohol | 10 | 44 | This study |
|  |  |  | p-cresol | 21 |  |  |
|  |  |  | phenol | 32 |  |  |

**Table S1. 3. Number of sites (amino acids) in the full and trimmed datasets filtered based on number of partitions and informativeness. Supermatrix M1 containing 2,638 orthologous sequence sets a; M2 with 1250 orthologous sequence sets; M3 with 625 orthologous sequence sets; M4 with 312 orthologous sequence sets; M5 with 739 orthologous sequence sets and informativeness score above 50; and M6 with the 312 orthologous sequence sets with highest informativeness.**

| **Dataset** | **M1** | **M2** | **M3** | **M4** | **M5** | **M6** |
| --- | --- | --- | --- | --- | --- | --- |
| Missing data (%) | 40 | 27 | 21 | 17 | 37 | 40 |
| Number of sites | 492,330 | 187,397 | 84,501 | 64,910 | 175,575 | 84,813 |

**Table S1.4. Divergence times, median dates and 95% highest posterior density (in millions of years), for all nodes across Diplopoda obtained from the BEAST analyses (18). Results are shown for both C1 and C2 calibrations.** For node numbers refer to supplemental Figure S1.1.

| **Node** | **Group** | **Median estimate C1** | **95% HPD C1** | **Median estimate C2** | **95% HPD C2** |
| --- | --- | --- | --- | --- | --- |
| 29 | Chilopoda + Diplopoda | 499 | 450-570 | 490 | 426-575 |
| 30 | Chilopoda | 420 | 417-453 | 390 | 384-399 |
| 31 | Diplopoda | 467 | 424-531 | 456 | 423-532 |
| 32 | Chilognatha | 426 | 423-467 | 412 | 339-485 |
| 33 | Pentazonia | 288 | 183-388 | 284 | 117-424 |
| 36 | Helminthomorpha | 349 | 285-402 | 359 | 311-420 |
| 37 | Colobognatha | 200 | 123-287 | 242 | 126-348 |
| 41 | Eugnatha | 290 | 265-383 | 325 | 307-374 |
| 45 | Juliformia | 234 | 163-302 | 215 | 106-311 |
| 52 | Merocheta | 178 | 200-322 | 128 | 139-341 |

**Table S1.5. Molecules present per family for each terminal taxon in Juliformia. We counted the number of molecules from the benzoquinone pathway present for the family represented by each terminal taxon and provide the source of chemical information (reference and/or this study). Isomers were counted as one. Missing data were coded as ?. Chemicals numbered as: [1] Phenol, [2] Hydroquinone, [3] 1,4-Benzoquinone, [4] 2-Methoxyhydroquinone, [5] 2-Methoxy-1,4-benzoquinone, [6] 2-Methoxy-3-hydroxy-1,4-benzoquinone, [7] 2,3-Dimethoxyhydroquinone, [8] 2,3-Dimethoxy-1,4-benzoquinone, [9] 2-Methylhydroquinone, [10] 2-Methyl-1,4-benzoquinone, [11] 2-Methyl-3-hydroxy-1,4-benzoquinone, [12] 2-Methoxy-3-methylhydroquinone, [13] 2-Methoxy-3-methyl-1,4-benzoquinone, [14] 2-Methoxy-3-methyl-5-hydroxy-1,4-benzoquinone, [15] 2-Methoxy-3,6-dimethylhydroquinone, [16] 2-Methoxy-3,6-dimethyl-1,4-benzoquinone, [17] 2,3-dimethoxy-5-methylhydroquinone, [18] 2,3-dimethoxy-5-methyl-1,4-benzoquinone, [19] 2-Ethyl-1,4-benzoquinone, [20] Naphthoquinone.**

| **Order** | **Family** | **Species** | **Molecules present in the family and source (either reference or this study)** | **Number of molecules** | **assuming presence of intermediates in pathway** |
| --- | --- | --- | --- | --- | --- |
| Julida | Paeromopodidae | *Californiulus_sp* | ? | ? Coded as minimum found (2) | ? |
|  | Nemasomatidae | *Orinisobates_nigrior* | 4, 5 (this study) | 2 | 4 |
|  | Parajulidae | *Uroblaniulus_sp* | 8 (this study) 10 (this study), 13 (6) | 2 | 4 |
|  | Julidae* | Julidae sp. | 1 (3,4,5), 3 (3,4,5), 5 (4,5), 6 (3), 7 (3,4,5), 8 (2,3,4,5), 9 (2,3,4,5), 10 (3,4,5), 11 (2,3,4,5), 12 (4), 13 (2,3,4,5), 14 (3), 17, 18, 19 | 16 | 16 |
| Spirobolida | Floridobolidae | *Floridobolus_sp* | 8 (9), 10 (8), 11 (7), 13 (8), 16 (9), 18 (9) | 6 | 13 |
|  | Rhinocricidae | *Anadenobolus_sp* | 2 (10), 3 (10), 7 (10), 8 (10), 9 (11,13), 10 (10,13), 11 (10), 12 (10,13), 13 (10,13), 16 (11), 17 (10), 18 (10,12), 19 (10), | 13 | 14 |
|  | Spirobolidae | *Narceus_sp* | 7 (15), 8 (15), 9 (this study), 10 (this study), 12 (15), 13 (14,15,this study), 18 (15) | 7 | 12 |
|  |  | *Tylobolus* | 7 (15), 8 (15), 9 (this study), 10 (this study), 12 (15), 13 (14,15,this study), 18 (15) | 7 | 12 |
|  | Spirobollelidae | *Microspirobolus_sp* | 4 (this study), 12 (this study), 13 (this study), 18 (this study) | 4 | 8 |
| Spirostreptida | Cambalidae | *Cambala_annulata* | 10 (16),13 (16) | 2 | 6 |
|  | Spirostreptidae | *Orthoporus_sp* | 2 (17), 3 (17), 4 (17), 5 (17), 7 (17), 8 (17), 9 (17), 10 (17), 12 (17), 13 (17), 18 (17), 19 (17), 20 (17) | 13 | 15 |
|  |  | *Orthoporus_sp* | 2, 3, 4, 5, 7, 8, 9, 10, 12, 13, 18, 19, 20 | 13 | 15 |

**Figure S1. 1. Divergence-time estimates based on 38 orthologous sequence sets in BEAST using calibration scheme C1. Median time estimates (in millions of years) and 95% highest posterior density intervals for each estimate are shown in Table S1.4.**

**Figure S1.2. Maximum likelihood ancestral character reconstruction for phenol and benzoquinone production as discrete characters. States are black (0): no phenol or benzoquinone production, grey (1): phenol production, red (2): benzoquinone production. Taxa with production of both chemicals were coded as polymorphic. A rate matrix was used with different rates for the following transformations: 0-1, 1-2 or 2-1, and 1 or 2-0.**

**Figure S1.3. Maximum likelihood ancestral character reconstruction for Heterocyclic nitrogen-cointaining compounds. States are white (0): absence, white (1): presence, grey. Equal rates (ER) is the model of character evolution with the highest likelihood score.**

**Figure S1.4. Maximum likelihood ancestral character reconstruction for Terpene compounds. States are white (0): absence, white (1): presence, grey. Equal rates (ER) is the model of character evolution with the highest likelihood score.**

**Figure S1.5. Maximum likelihood ancestral character reconstruction for cyanogenic compounds. States are white (0): absence, grey (1): presence, red. Equal rates (ER) is the model of character evolution with the highest likelihood score.**

**References**

1. Brewer MS, Bond JE (2013) Ordinal-level phylogenomics of the arthropod class Diplopoda (millipedes) based on an analysis of 221 nuclear protein-coding loci generated using next-generation sequence analyses. *PLoS One* 8(11):e79935.

2. Bodner M, Raspotnig G (2012) Millipedes that smell like bugs: (E)-Alkenals in the defensive secretion of the julid diplopod Allajulus dicentrus. *J Chem Ecol* 38(5):547–556.

3. Bodner M, et al. (2016) “Quinone millipedes” reconsidered: Evidence for a mosaic-like taxonomic distribution of phenol-based secretions across the Julidae. *J Chem Ecol* 42(3):249–258.

4. Makarov SE, et al. (2017) Chemical ecology of cave-dwelling millipedes: Defensive secretions of the Typhloiulini (Diplopoda, Julida, Julidae). *J Chem Ecol* 43(4):317–326.

5. Sekulić T, et al. (2014) Quinones and non-quinones from the defensive secretion of Unciger transsilvanicus (Verhoeff, 1899) (Diplopoda, Julida, Julidae), from Serbia. *Arch Biol Sci* 66(1):385–390.

6. Shear WA (2015) The chemical defenses of millipedes (Diplopoda): Biochemistry, physiology and ecology. *Biochem Syst Ecol* 61:78–117.

7. Kluge AF, Eisner T (1971) Defense mechanisms of arthropods. XXVIII. A quinone and a phenol in the defensive secretion of a parajulid millipede. *Ann Entomol Soc Am* 64:314–315.

8. Weatherston J, Percy JE (1969) Studies of physiologically active arthropod secretions. III. Chemical, morphological, and histological studies of the defence mechanism of Uroblaniulus canadensis (Say) (Diplopoda:Julida). *Can J Zool* 47(6):1389–1394.

9. Attygalle AB, Xu S-C, Meinwald J, Eisner T (1993) Defensive secretion of the millipede Floridobolus penneri. *J Nat Prod* 56(10):1700–1706.

10. Wu X, Buden DW, Attygalle AB (2007) Hydroquinones from defensive secretion of a giant Pacific millipede, Acladocricus setigerus (Diplopoda: Spirobolida). *Chemoecology* 17(3):131–138.

11. Buden DW, Attygalle AB, Wu X (2004) Distribution of the Chuuk Islands giant millipede, Acladocricus setigerus (Spirobolida: Rhinocricidae), and identification of its defensive compounds. *Pacific Sci* 4(2004):625–636.

12. Arab A, et al. (2003) Composition of the defensive secretion of the Neotropical millipede Rhinocricus padbergi Verhoeff 1938. *Entomotropica* 18:79–82.

13. Bedoussac L, Favila ME, López RM (2007) Defensive volatile secretions of two diplopod species attract the carrion ball roller scarab Canthon morsei (Coleoptera: Scarabaeidae). *Chemoecology* 17(3):163–167.

14. Percy JE, Weatherston J (1971) Studies of physiologically active arthropod secretions. V. Histological studies of the defence mechanism of Narceus annularis (Raf.) (Diplopoda: Spirobolida). *Can J Zool* 49(2):278–279.

15. Kuwahara Y, Noguchi S, Mori N, Higa Y (2002) Identification of benzoquinones and hydroquinones as the secretory compounds from three species of Okinawan millipedes. *Japanese J Environ Entomol Zool* 13(3):117–124.

16. Eisner T, Hurst JJ, Keeton WT, Meinwald Y (1965) Defense mechanisms of arthropods. XVI. Para-benzoquinones in the secretion of spirostreptoid millipedes. *Ann Entomol Soc Am* 58:247–248.

17. Deml R, Huth A (2000) Benzoquinones and hydroquinones in defensive secretions of tropical millipedes. *Naturwissenschaften* 87:80–82.

18. Drummond A, Rambaut A (2007) BEAST: Bayesian evolutionary analysis by sampling trees. *BMC Evol Biol* 7(1):214.
